# Supplementary material for: Antimicrobial activity of Lactobacillus spp. isolated from fermented foods and their inhibitory effect against foodborne pathogens
Source: PeerJ. 2025 Jan 6;13:e18541. doi: 10.7717/peerj.18541 (PMC11716013; doi:10.7717/peerj.18541)
Supplement: Supplemental Information 1 — Colony counts and calculated CFU/mL values are provided for each sample, with the green olives pickle sample having colonies too numerous to count (TMTC). These results demonstrate varying microbial densities across the food sources, highlighting differences in microbial populations associated with each type of fermentation. [file peerj-13-18541-s001.docx]

**Table a**. cfu/mL of *Lactobacillus* spp. grown on MRS agar after 48 h

| **Sample** | **Source** | **Number of colonies** | **CFU/ml** |
| --- | --- | --- | --- |
| 1 | yoghurt | 88 | 88 ×10^5^ |
| 2 | Feta cheese | 62 | 62×10^5^ |
| 3 | Kafir cheese | 57 | 57×10^5^ |
| 4 | Turkey cheese | 75 | 75×10^5^ |
| 5 | Black olives pickle | 47 | 47×10^5^ |
| 7 | Green olives pickle | TMTC | TMTC |
| 8 | Cucumber pickle | 50 | 50×10^5^ |
