# Supplementary material for: Antimicrobial activity of Lactobacillus spp. isolated from fermented foods and their inhibitory effect against foodborne pathogens
Source: PeerJ. 2025 Jan 6;13:e18541. doi: 10.7717/peerj.18541 (PMC11716013; doi:10.7717/peerj.18541)
Supplement: Supplemental Information 9 — The process begins with the collection of food samples, followed by sample preparation, pre-enrichment in MRS broth, and plating on MRS agar for the isolation and confirmation of Lactobacillus spp. Morphological observation, biochemical tests, and 16S rRNA sequencing identify L. plantarum KR3 as the strain of interest. The antimicrobial potential of L. plantarum KR3 is assessed using the well-diffusion method, demonstrating significant inhibition against foodborne pathogens. Further investigations evaluate the effect of pH, temperature, and enzyme treatments on the strain’s activity. Additionally, the anti-biofilm activity of L. plantarum KR3 cell-free supernatant (CFS) is analyzed, showing effective biofilm disruption at 1x MIC concentration when compared to the control.This study highlights the potential application of L. plantarum KR3 in combating biofilms and foodborne pathogens, emphasizing its role in food safety and preservation. [file peerj-13-18541-s009.pdf]

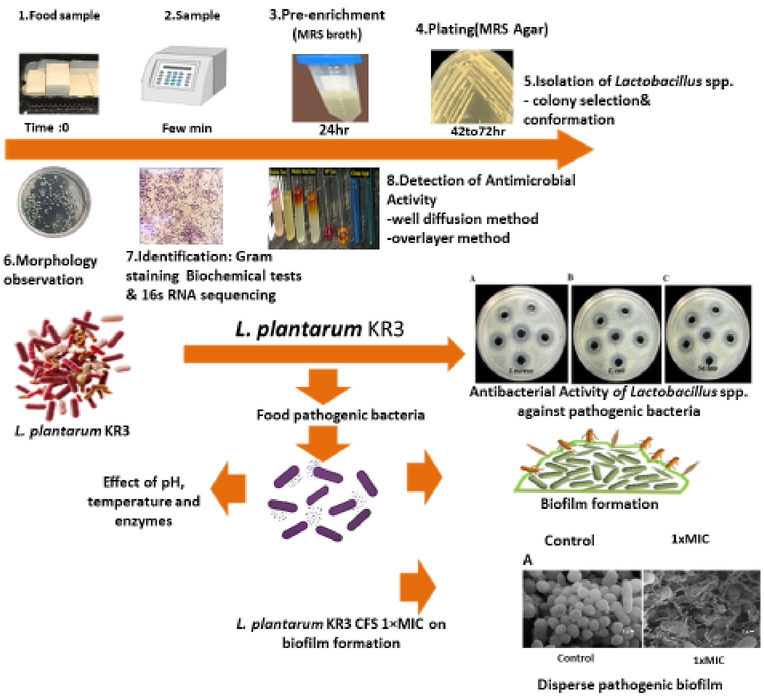

1.Food sample
2.Sample
3.Pre-enrichment (MRS broth)
4.Plating(MRS Agar)
5.Isolation of Lactobacillus spp.
- colony selection&
conformation
Time :0
Few min
24hr
42to72hr
8.Detection of Antimicrobial Activity
-well diffusion method
-overlayer method
6.Morphology observation
7.Identification: Gram staining Biochemical tests & 16s RNA sequencing
L. plantarum KR3
A
B
C
Antibacterial Activity of Lactobacillus spp. against pathogenic bacteria
L. plantarum KR3
Food pathogenic bacteria
Effect of pH, temperature and enzymes
Biofilm formation
Control
1xMIC
A
L. plantarum KR3 CFS 1×MIC on biofilm formation
Control
1xMIC
Disperse pathogenic biofilm

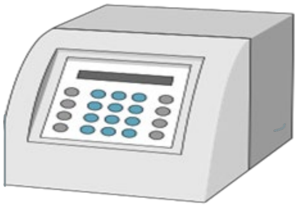

Image: File:202406 Homogenizer.svg by DataBase Center for Life Science (DBCLS), used under CC BY 4.0. Modified by Athraa. Original source: DOI https://doi.org/10.7875/togopic.2024.147.

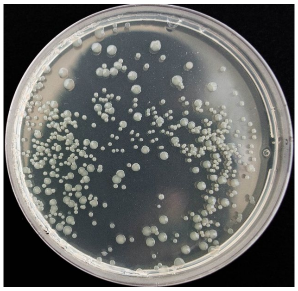

Image by Nadya il from Pixabay, used under the Pixabay license.

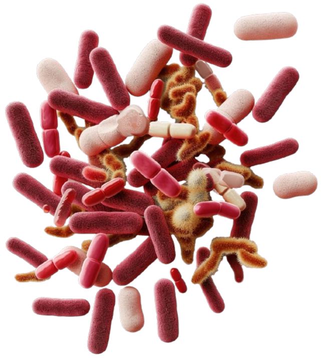

Image of Lactobacillus and Bifidobacterium by PNGTree, used under license.

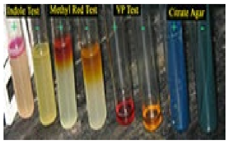

The IMViC Results image originates from work conducted at Acharya Prafulla Chandra College, New Barrackpur, West Bengal, India. It is licensed under CC BY-SA 3.0 via Wikimedia Commons. https://w.wiki/CKLF
